# KinFin: Software for Taxon-Aware Analysis of Clustered Protein Sequences

**DOI:** 10.1534/g3.117.300233

**Published:** 2017-09-01

**Authors:** Dominik R. Laetsch, Mark L. Blaxter

**Affiliations:** * Institute of Evolutionary Biology, University of Edinburgh, EH9 3JT, United Kingdom; † The James Hutton Institute, DD2 5DA Dundee, United Kingdom

**Keywords:** bioinformatics, protein orthology, protein family evolution, comparative genomics, filarial nematodes

## Abstract

The field of comparative genomics is concerned with the study of similarities and differences between the information encoded in the genomes of organisms. A common approach is to define gene families by clustering protein sequences based on sequence similarity, and analyze protein cluster presence and absence in different species groups as a guide to biology. Due to the high dimensionality of these data, downstream analysis of protein clusters inferred from large numbers of species, or species with many genes, is nontrivial, and few solutions exist for transparent, reproducible, and customizable analyses. We present KinFin, a streamlined software solution capable of integrating data from common file formats and delivering aggregative annotation of protein clusters. KinFin delivers analyses based on systematic taxonomy of the species analyzed, or on user-defined, groupings of taxa, for example, sets based on attributes such as life history traits, organismal phenotypes, or competing phylogenetic hypotheses. Results are reported through graphical and detailed text output files. We illustrate the utility of the KinFin pipeline by addressing questions regarding the biology of filarial nematodes, which include parasites of veterinary and medical importance. We resolve the phylogenetic relationships between the species and explore functional annotation of proteins in clusters in key lineages and between custom taxon sets, identifying gene families of interest. KinFin can easily be integrated into existing comparative genomic workflows, and promotes transparent and reproducible analysis of clustered protein data.

Inference of gene homology across taxa is an integral part of comparative genomic analysis. Candidate orthologs, paralogs, and xenologs between taxa are commonly identified through clustering of protein sequence data using tools such as OrthoFinder (Emms and Kelly 2015), OrthoMCL (Li *et al.* 2003), and others. Exploitation of ortholog definitions across species, the study of gene family evolution, genome evolution, species phylogenetics, and as loci for population genetics and ecological genetics, is demanding. Many research projects aim to identify orthologs of interest that have a specific distribution across species, for example, identifying gene families that are synapomorphic for, or that have been specifically lost from, a particular clade. Exploring the effects of assuming different underlying phylogenies on the analysis of the origins of orthologs may assist in discriminating between competing hypotheses. Grouping species by nonphylogenetic classifiers (such as habitat, mating system, or life history) may also identify protein families uniquely present/absent or exhibiting differential copy-number. Several high-quality solutions to orthology analysis have been proposed. OrthoDB is a high-quality curated orthology resource (Zdobnov *et al.* 2017). The current release (2015) includes 3600 bacterial and 590 eukaryotic taxa, and is accessed through a responsive web interface or direct download and interrogation. OrthoDB includes rich functional annotation of sequences. While the main database includes only published genomes, and is centrally managed (*i.e.*, users cannot submit datasets for analysis), the OrthoDb software toolkit is available for local installation and deployment. PhylomeDB is a database of defined orthology groups, built with manual curation (Huerta-Cepas *et al.* 2014), but was last updated in 2014, and is, again, managed centrally and focused on published genomes. In the ENSEMBL databases, the Compara toolkit is used to parse gene homology sets, and infers orthology and paralogy based on a given species tree (Herrero *et al.* 2016). Updating of Compara analyses is not trivial, and requires the ENSEMBL web toolkit for display and interrogation. For ongoing research programs, few tools exist for orthology analysis. For bacterial data, several tools for pan-genome analysis have been developed (Vinuesa and Contreras-Moreira 2015; Chaudhari *et al.* 2016; Xiao *et al.* 2015) but solutions that cope well with the data richness of eukaryotic species are often tailored to defined taxonomic groups (Song *et al.* 2015) or expect closely related taxa. EUPAN is a pipeline for pan-genome analysis of closely related eukaryotic genomes developed within the scope of the 3000 Rice Genomes Project (Hu *et al.* 2017). The approach parts from mapping of raw reads to reference genomes followed by coordinated assembly and lift-over of gene annotations for inferring presence/absence of gene models. In the absence of toolkits that allow local implementation of clustering analyses, custom taxon grouping and dynamic analysis, we have developed KinFin. KinFin takes a protein clustering output by tools such as OrthoFinder or OrthoMCL, alongside functional annotation data, and user-defined species taxonomies, to derive rich aggregative annotation of orthology groups. KinFin reads from standard file formats produced from commonly used genome sequencing and annotation projects. KinFin can easily be integrated in comparative genomics projects for the identification of protein clusters of interest in user-defined, taxon-aware contexts.

## Methods

KinFin is a standalone Python 2.7 application. A detailed description of the functionality of KinFin can be found at https://kinfin.readme.io/. Required input for KinFin is an orthology clustering (format defined by OrthoMCL/OrthoFinder), a file linking protein sequences to taxa (SequenceIDs defined by OrthoFinder), and a user-defined config file. The config file guides analyses by grouping taxa into user-defined sets under arbitrary attributes. These attributes could include, for instance, standard taxonomy (as embodied in the NCBI Taxonomy “TaxID” identifiers), alternate systematic arrangements of the taxa involved, lifestyle, geographical source, or any other aspect of phenotype or other metadata. KinFin dynamically constructs sets based on the config file and computes metrics, statistics, and visualizations, which allows identification of clusters that are diagnostic for, or expanded/contracted in, each taxon set. Optional input files include proteome FASTA files (to extract length statistics for clusters, taxa and taxon sets), functional annotations of proteins in InterProScan (Jones *et al.* 2014) format, and a phylogenetic tree topology in Newick format.

### Visualization of ortholog clustering

**Figure 1:**
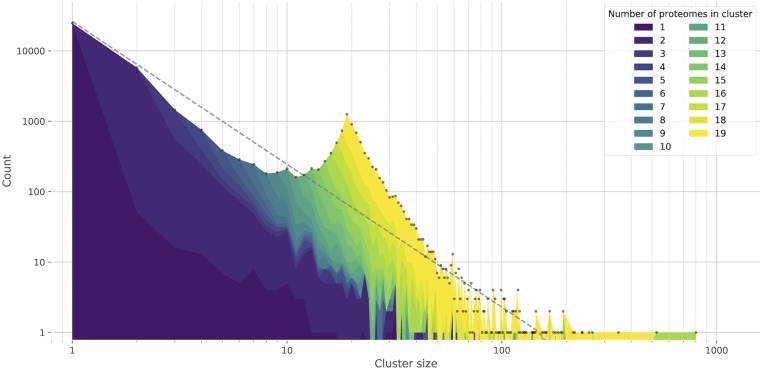
Distribution of cluster sizes in clustering. The distribution of counts of proteins per cluster is colored based on the number of proteomes present. Total values of counts of each cluster size are indicated with gray dots. A fitted power-law curve (gray) is drawn for reference.

In KinFin, global analysis of the clustering of protein sequences can be performed from the perspective of the clusters themselves (their properties and patterns) or of the constituent proteomes. The distribution of cluster size (*i.e.*, the number of proteins contained in a cluster) is an important feature of analyses, and KinFin simplifies the comparison of alternative clusterings (for example ones using different MCL inflation parameters, or with overlapping but distinct taxon inclusion) by generating frequency histograms of cluster size, which can be interrogated for deviations from the expected power law-like distribution. To aid understanding of the distribution, the user can generate a more detailed frequency histogram which considers the number of taxa contributing to each cluster (Figure 1). The behavior of individual proteomes can be explored by creating a network representation of the clustering. KinFin can produce a graph file with nodes representing proteomes and edges connecting nodes weighted by the number of times two proteomes co-occur in clusters. Optionally, universal clusters with members from all the proteomes can be excluded. The graph can be interrogated using graph analysis and visualization tools such as Gephi (Bastian *et al.* 2009) (see Supplemental Material, Figure S1 in File S1).

### Analyses based on arbitrary sets of input proteomes

Through the config file, the user can instruct KinFin to analyze the clustering under arbitrary sets. For taxonomy-based analyses, KinFin derives analyses at different taxonomic ranks (by default “phylum,” “order,” and “genus”; can be modified by the user) by parsing the NCBI TaxIDs given for each proteome. Any other classification of the input proteomes can be given, and nested taxonomies specified by use of multiple, ranked attribute types. This allows, for example, the testing of congruence of clustering data with competing phylogenetic hypotheses regarding relationships of the taxa from which the input proteomes were derived.

### Classification of clusters

**Figure 2:**
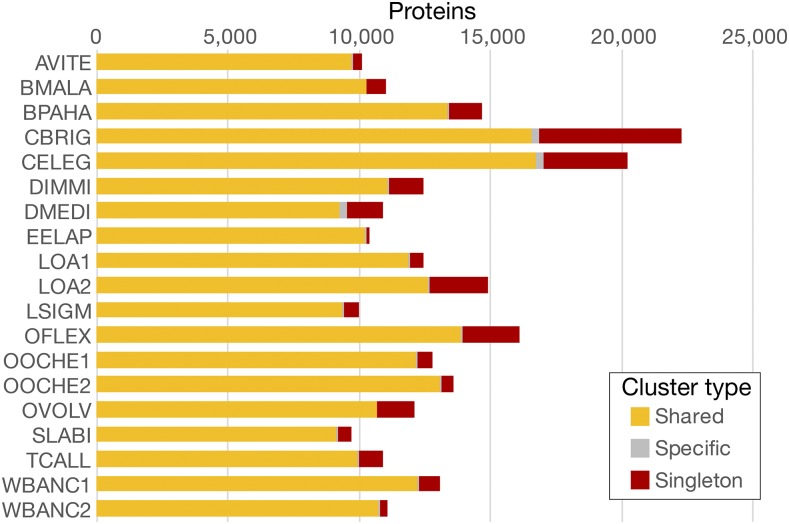
Count of proteins by type of cluster. “Shared”: clusters containing proteins from multiple taxa, “Specific”: clusters containing two or more proteins from a single proteome, “Singleton”: clusters containing a single protein.

KinFin builds a series of matrices associating clusters and proteomes, and clusters and arbitrary proteome sets. Each cluster is classified as “absent” or “present” for each proteome or taxon set, and is assigned a cluster type (“singleton,” “specific” - where there are ≥2 members and all come from one taxon set - or “shared”) (Figure 2).

### Single-copy ortholog definition

**Figure 3:**
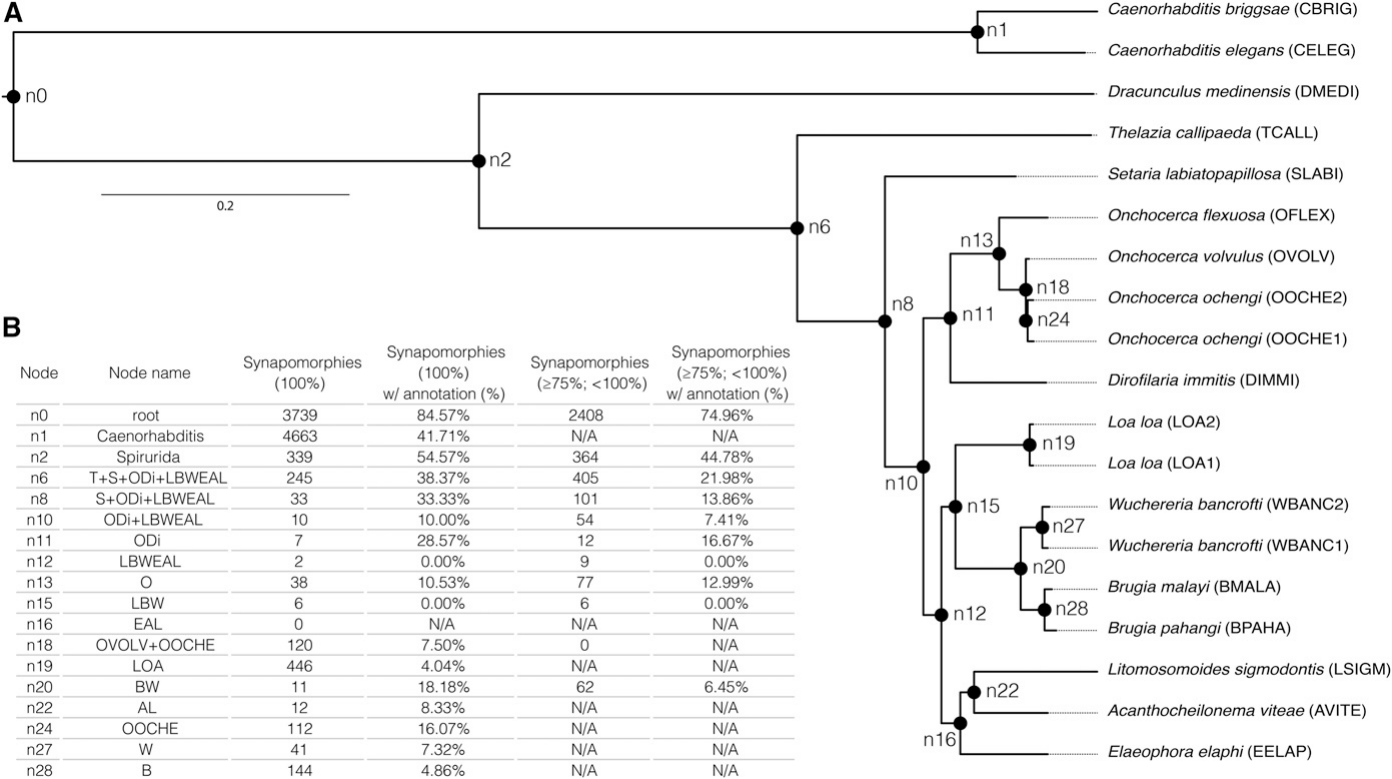
(A) Phylogenetic tree based on 781 single-copy orthologs. Nonparametric bootstrap support for all branches is 100. Internal nodes are labeled. (B) Table summarizing “complete presence” synapomorphic clusters (100% taxon-coverage) and “partial absence” synapomorphic clusters (75% ≤ taxon-coverage < 100%), and the percentage for which a representative functional annotation could be inferred. “N/A” is used for cases in which nodes are ancestors of more than four taxa, or when percentage of functional annotation could not be calculated due to lack of clusters.

Clusters composed of a single protein from each proteome (*i.e.*, putative single-copy orthologs) are useful for downstream phylogenetic analyses (Figure 3). However, due to the intrinsic difficulties of genome assembly and annotation, the number of single-copy orthologs decreases the more proteomes are included in the clustering. To compensate for this, KinFin can identify “fuzzy” single-copy ortholog clusters using the parameters target_count (target number of copies per proteome, default “1”), target_fraction (proportion of proteomes at “target number”), and lower/upper bounds for proteomes outside of target_fraction (min and max).

### Rarefaction curves

**Figure 4:**
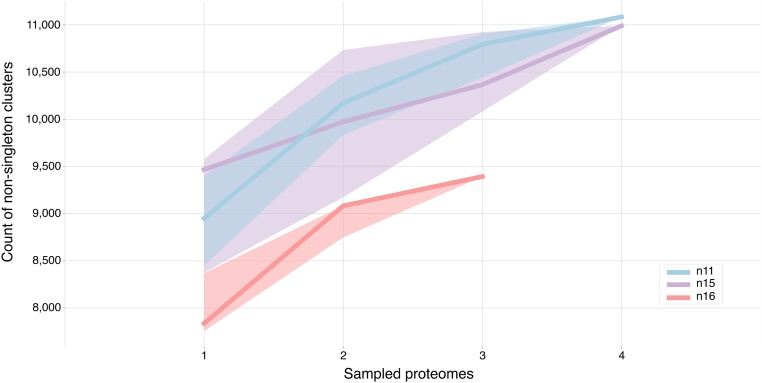
Rarefaction curve of proteomes within sets defined by major clades within the onchocercid nematodes. n11, node 11 (*D. immitis* and *Onchocerca* species); n15, node 15 (*W. bancrofti*, *Brugia* species and *L. loa*); n16, node 16 (*L. sigmodontis*, *A. viteae*, and *E. elaphi*).

The concept of the pangenome is frequently used in microbial genomics to describe all the genes, both core and accessory, that are found in the varied genomes of a species. The size of the pangenome can be visualized using rarefaction curves, and KinFin deploys this framework to visualize the size of the pan-proteome of the different arbitrary sets defined by the user. Curves are calculated by repeated, random sampling of the proteomes in each arbitrary set, and cumulative summation of novel nonsingleton clusters (Figure 4).

### Pairwise protein count representation tests

**Figure 5:**
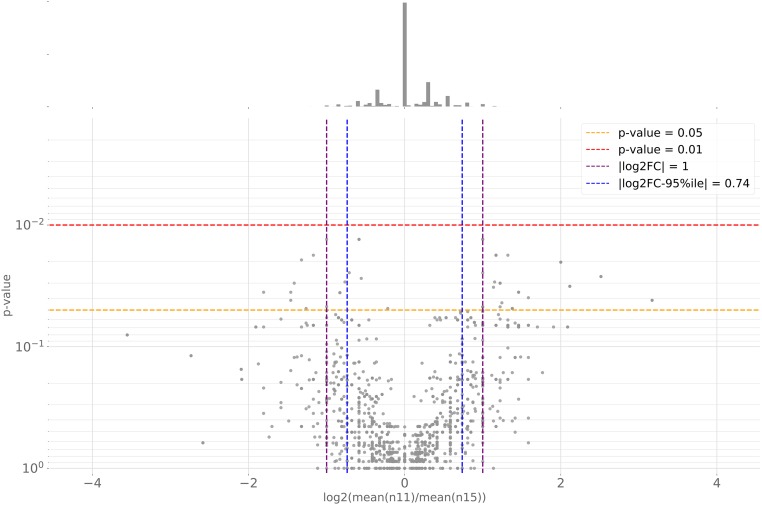
Volcano plot of the protein count representation tests for clusters shared between taxa at node n11 (*D. immitis* and *Onchocerca* species) and node n15 (*W. bancrofti*, *Brugia* species, and *L. loa*).

For user-defined attributes involving two (or more) taxon sets, pairwise representation tests of protein counts are computed for clusters containing proteins from each taxon set using either two-sided Mann-Whitney *U*-tests (default), Welch’s *t*-tests, or Student’s *t*-tests. From this, clusters “enriched” or “depleted” in count in one set compared with another can be identified. It should be noted that these statistical tests test for nonhomogeneity of the distributions of protein counts between the sets, and, due to limited “sample size,” might not achieve significance even when counts differ substantially between sets. In addition to text outputs, volcano plots ($\log_{2}$-fold change in means *vs.* test *P*-value) are drawn (Figure 5). As a visual aid, horizontal lines are drawn at *P*-values 0.05 and 0.01 and vertical lines at $\left|\log_{2}\text{-fc}\left(\text{means}\right)\right|$ = 1 and 95%-percentile of $\log_{2}$-fold changes in mean.

### Analyses based on functional annotation and protein length data

KinFin integrates functional annotation and protein length data into analyses. If the necessary input files are provided, KinFin generates output files tabulating mean and SD of sequence lengths, domain, and Gene Ontology (GO) term entropy within clusters, and the fraction of proteins per cluster which are putatively secreted, based on “SignalP_Euk” (Petersen *et al.* 2011) annotation. Additionally, for each cluster, all matching domains and inferred GO terms are listed with description and information regarding their frequency within both proteins and proteomes in the cluster.

While inference of functional annotation of a protein is relatively straightforward, no clear standards exist for inferring representative functional annotation of clusters of proteins. We provide a script with parameters domain_taxon_cov (minimum fraction of taxa in cluster that have at least one protein annotated with that domain) and domain_protein_cov (minimum fraction of proteins in cluster annotated with that domain), to grant users fine control over cluster functional annotation.

### Analyses based on phylogeny

Analysis of clusters in a phylogenetic context permits the identification and quantification of clusters that are unique innovations of certain monophyletic groups (*i.e.*, synapomorphies). Based on a user-defined tree topology, KinFin identifies synapomorphic clusters at nodes using Dollo parsimony. The Dollo parsimony method (Farris 1977) assumes that while multiple independent losses of a gene in different lineages are common, multiple independent gains of the same gene are improbable. The implementation of Dollo parsimony in KinFin requires that only the proteomes under a given node are members of the cluster, and that at least one taxon from each child node is a member. Because Dollo parsimony does not penalize multiple losses, KinFin classifies synapomorphies into “complete presence” and “partial absence” subgroups. The outputs include lists of synapomorphies and apomorphies (“singleton” and “nonsingleton” proteome-specific clusters), and detailed description of synapomorphic clusters at each node. Prominent or consistent functional annotation can be mapped onto synapomorphic clusters, filtered by node_taxon_cov (minimum presence of proteomes as fraction of total proteomes under the node), domain_taxon_cov (minimum proportion of proteomes represented by proteins with a specific domain annotation), and domain_protein_cov (minimum proportion of all the proteins annotated with a specific domain).

### Analyses of clusters containing genes of interest

Output of protein clustering analysis often serves as substrate for the identification of homologs of genes of interest from a model species in the target species. KinFin is distributed with a script that takes as input a list of protein IDs or of gene IDs (to obtain all isoforms or only the isoforms included in the clustering), and writes tables indicating the counts of proteins in each cluster and their representative functional annotations.

### Output

KinFin generates output folders for each relevant column in the config file, and writes overall metrics for all taxon sets, detailed metrics for each cluster and results of pairwise representation test, draws the rarefaction curve and volcano plots, and lists clusters classified as “true” and “fuzzy” single-copy orthologs. Resulting text files can easily be interrogated using common UNIX command line tools or spreadsheet software.

### Operation

KinFin is freely available under GNU General Public License v3.0 at https://github.com/DRL/kinfin. System requirements for KinFin include a UNIX-based operating system, Python 2.7, and pip. An installation script is provided, which installs Python dependencies and downloads mapping files for Pfam, Interpro and GO IDs from the European Bioinformatics Institute (EBI) website. Instructions for installation and execution of KinFin can be found on the GitHub repository and detailed documentation is available at https://kinfin.readme.io.

### KinFin in action: filarial nematodes parasitic in humans and other vertebrates

We illustrate some of the main functionalities of KinFin by addressing questions regarding the biology of filarial nematodes. Filarial nematodes (Onchocercidae) include many species of medical and veterinary interest, and the phylogenetic relationships among them remain under debate (Park *et al.* 2011; Nadler *et al.* 2007), with the current NCBI reference taxonomy likely to be incorrect. We analyzed the proteomes of 16 species: 11 filarial nematodes, three related spirurid nematodes and two *Caenorhabditis* species. *Caenorhabditis* were included because of the quality of available structural and functional annotations. For three species, two independent assemblies and proteome predictions were included.

We will use KinFin to generate a robust multi-locus alignment and phylogeny, and then incorporate this tree into KinFin analyses of synapomorphies and other features of groups of filaria. What gene family and functional differences characterize the different groups? Finally, we will use sets of *Caenorhabditis elegans* genes implicated in pathways of interest to highlight orthology and paralogy in the filarial nematodes.

### KinFin analysis protocol

Here, we summarize the protocol used to generate the analyses reported. Detailed protocols including program versions and command lines are given in Supplemental Methods in File S1.

The proteomes detailed in Table 1 were downloaded from WormBase parasite (WBPS8) (Howe *et al.* 2016, 2017) and http://ngenomes.org. For currently unpublished genomes, manuscripts giving details of assembly and annotation strategies are in preparation. The files were filtered by keeping only the longest isoforms and excluding sequences <30 residues or containing nonterminal stops. Proteins were functionally annotated using InterProScan (Jones *et al.* 2014), and clustered via OrthoFinder (Emms and Kelly 2015) at default MCL inflation value of 1.5 using BLASTp (Camacho *et al.* 2009) similarity data.

**Table 1:** Proteomes used in OrthoFinder clustering and subsequent KinFin analyses

Taxon ID*^a^*	Species Name	Source	Version	Proteins*^b^*
AVITE	*A. viteae*	WBPS8	PRJEB4306	10,123
BMALA	*B. malayi*	WBPS8	PRJNA10729	11,008
BPAHA	*B. pahangi*	WBPS8	PRJEB497	14,664
CBRIG	*C. briggsae*	WBPS8	PRJNA10731	22,305
CELEG	*C. elegans*	WBPS8	PRJNA13758	20,219
DIMMI	*D. immitis*	WBPS8	PRJEB1797	12,423
DMEDI	*D. medinensis*	WBPS8	PRJEB500	10,919
EELAP	*E. elaphi*	WBPS8	PRJEB502	10,409
LOA1	*L. loa*	WBPS8	PRJNA246086	12,473
LOA2	*L. loa*	WBPS8	PRJNA60051	14,908
LSIGM	*L. sigmodontis*	WBPS8	PRJEB3075	10,001
OFLEX	*O. flexuosa*	WBPS8	PRJEB512	16,094
OOCHE1	*O. ochengi*	WBPS8	PRJEB1204	12,807
OOCHE2	*O. ochengi*	WBPS8	PRJEB1809	13,580
OVOLV	*O. volvulus*	WBPS8	PRJEB513	12,110
SLABI	*S. labiatopapillosa*	ngenomes.org	nSl.1.1	9,687
TCALL	*T. callipaeda*	WBPS8	PRJEB1205	10,911
WBANC1	*W. bancrofti*	WBPS8	PRJEB536	13,056
WBANC2	*W. bancrofti*	WBPS8	PRJNA275548	11,053

a ID used in KinFin analysis.

b Proteins included in the clustering analysis.

An initial KinFin analysis identified 781 one-to-one single-copy orthologs. Sequences for these 781 clusters were extracted and aligned using mafft (Katoh and Standley 2013). Alignments were trimmed using trimal (Capella-Gutiérrez *et al.* 2009), concatenated using FASconCAT (Kück and Meusemann 2010), and analyzed using RAxML (Stamatakis 2014) under the PROTGAMMAGTR model of sequence evolution. Nonparametric bootstrap analysis was carried out for 100 replicates.

KinFin was then rerun, providing additional classification in the config file and functional annotation data (Supplemental Dataset 2). The topology of the tree inferred through phylogenetic analysis was provided in Newick format, and the two *Caenorhabditis* species were specified as outgroups. The Mann-Whitney *U*-test was selected for pairwise protein count representation tests. Representative functional annotation was inferred for all clusters, and analyzed specifically for subsets of taxa. Genes involved in heme biosynthesis and homeostasis were identified based on representatives from *C. elegans*, and absence of missing genes was confirmed using TBLASTn (Camacho *et al.* 2009) against the respective genomes. Presence of paralogs was confirmed by manual inspection of gene models on WormBase ParaSite.

### Data availability

Supplemental Material from this study is deposited at https://zenodo.org under 10.5281/zenodo.844364.

Supplemental_data_1.tar.gz contains input files for basic KinFin analysis.
Supplemental_data_2.tar.gz contains input files for advanced KinFin analysis.
Supplemental_data_3.tar.gz contains results of KinFin analyses.

## Results

The 19 proteomes (derived from 16 species) included 248,750 protein sequences (with a total amino-acid length of 95,162,557 residues) after filtering. OrthoFinder places these into 42,691 clusters, of which 57.97% were singletons (containing 9.95% of protein sequences). The clusters displayed a power-law-like frequency distribution, but with a marked deviation from this expectation at a cluster size of 19 (Figure 1). This pattern, although less pronounced, has been observed before for protein databases such as COG (Clusters of Orthologous Groups of proteins) (Unger *et al.* 2003) and TRIBES (Enright *et al.* 2003), and has been seen in other datasets analyzed with KinFin (Yoshida *et al.* 2017). These clusters contain a large number of strict (“true”) single-copy orthologs, and many “fuzzy” single-copy orthologs.

KinFin can assist in deciding which of several alternative proteome predictions is more likely to be correct, and identify major differences between alternative predictions. Analysis of between-proteome linkage using Gephi (see Supplemental Results in File S1) showed that while some proteome pairs (for example, the two from *Onchocerca ochengi*) were found to be neighbors, the two *Wuchereria bancrofti* proteomes were not placed together, suggesting divergence in prediction. Examination of the distribution of clusters within species can highlight outlier datasets. Both *C. elegans* and *Caenorhabditis briggsae* have higher total protein counts than any of the filarial species, and display the highest proportion of singletons (15.7 and 24.4%) (Figure 2). For those species for which two assemblies were analyzed, variation in proportion of singletons is most severe for *Loa loa* (15.1% *vs.* 4.6%).

The 781 single-copy orthology clusters yielded a robustly supported phylogeny (Figure 3A). Rooting the tree with *Caenorhabditis*, the three nonfilarial taxa are recovered in expected positions, with *Setaria labiatopapillosa* most closely related to the onchocercids, followed by *Thelazia callipaeda* and *Dracunculus medinensis*. The relationships between the onchocercid taxa is not congruent with the reference NCBI taxonomy, but, with a previous analysis using a smaller number of loci (Lefoulon *et al.* 2016). *Dirofilaria immitis* is recovered as sister to *Onchocerca* species (the clade defined by node n11 in Figure 3A), and *W. bancrofti*, *Brugia* species and *L. loa* (node n15) form a clade distinct from *Litomosomoides sigmodontis*, *Acanthocheilonema viteae* and *Elaeophora elaphi* (node n16). We identified 3887 “fuzzy” single-copy orthologs. These are useful for analyses of proteomes from more distantly related species, where stochastic absence and duplication can severely limit the number of single-copy loci recovered for phylogenetic analyses. “Fuzzy” orthologs can be used in combination with tools such as PhyloTreePruner (Kocot *et al.* 2013), which filters out-paralogs and selects appropriate in-paralogs.

We explored the proteomic diversity represented by the three clades within Onchocercidae (Figure 3A, at nodes n11, n15, and n16). We defined taxon sets under each of the nodes, and used KinFin to generate rarefaction curves for each set (Figure 4). Curves for node n11 (*D. immitis* and *Onchocerca* species) and node n15 (*W. bancrofti*, *Brugia* species, and *L. loa*) show comparable slopes, and the number of nonsingleton clusters recovered in both is very similar (11,084 clusters for n11 and 10,989 for n15). Fewer unique protein clusters (9393) were recovered when sampling node n16 (*L. sigmodontis*, *A. viteae*, and *E. elaphi*). The fact that none of the curves reaches a plateau suggests that their protein space has not been sampled exhaustively.

Of the proteins used in the analysis, 157,873 (63.47%) were annotated with InterPro (IPR) domains. By inferring representative IPR annotations, we functionally annotated 12,026 (28.17%) of the clusters. Using the phylogeny inferred from single-copy orthologs (Figure 3A) we inferred synapomorphies at each node and investigated their representative functional annotations (Figure 3B). While many clusters are inferred to be synapomorphic for deeper nodes, Onchocercidae and the three groups within Onchocercidae have few synapomorphic gene family births (10 at node n10, seven at node n11, six at node n15 and none at node n16). Of those, only two clusters at node n11 received representative functional annotation: a Chromadorea ALT cluster (cluster “OG0007060”) and a SOCS box domain cluster (“OG0009843”). The Chromadorea ALT domain is found across Nematoda and in several clusters. *Brugia malayi* ALT-1, the first described Chromadorea ALT protein (contained in cluster “OG0000082”), has been proposed as a candidate vaccine target for human lymphatic filariasis (Gregory *et al.* 2000). The synapomorphic Chromadorea ALT cluster is specific to *Onchocerca* spp. and *D. immitis*, and might harbor the same potential for onchocerciasis. SOCS box domains were first identified in proteins involved in suppression of cytokine signaling, and are key regulators of both innate and adaptive immunity (Alexander 2002). Proteins in “OG0009843” do not contain any of the other domains usually associated with SOCS, such as SH2 (a combination found in “OG0000874” and “OG0007539”) or Ankyrin repeat-containing domains (a combination found in “OG0001559” and “OG0015826”). However, they may still play an immunomodulatory role during infection, as has been suggested for SOCS box proteins in *L. sigmodontis* (Godel *et al.* 2012). An additional SOCS box cluster is synapomorphic for *Onchocerca* species.

Definition of taxon sets based on host species (“human” *vs.* “other” *vs.* “outgroup”) recovered 628 clusters specific to filarial nematodes, but none had proteins of more than four out of seven taxa. Of the eight clusters containing proteins of four taxa, six contained only *B. malayi*, *L. loa*, and *W. bancrofti*. Hence, we found no evidence of systematic convergent adaptation to human hosts in the analyzed proteomes of filarial nematodes. KinFin permits rapid assessment of differences in copy number between species and taxon sets using protein count representation tests. Analysis of clusters shared between taxa either side of the basal split in Onchocercinae (node n11: *Dirofilaria* plus *Onchocerca*, and node n15: other filaria) (Figure 5) identified 10 clusters with extreme differences (see Table S1 in File S1). Among these was cluster “OG0000051,” which includes prolyl 4-hydroxylase orthologs, including Bm-PHY-1 and Bm-PHY-2, which are essential for development and cuticle formation, and have been suggested as potential targets for parasite control (Winter *et al.* 2013). While all n15 taxa have exactly two paralogs (WBANC2 had only Wb-PHY-1, but Wb-PHY-2 was located through a TBLASTN search and was present in WBANC1), counts in n11-taxa ranged from five (OFLEX) to 14 (OVOLV). We identified three additional, singleton prolyl 4-hydroxylase clusters, all from n15 taxa. The number of paralogous prolyl 4-hydroxylases in *D. immitis* and *Onchocerca* species could have negative implications in control measures against this locus.

**Figure 6:**
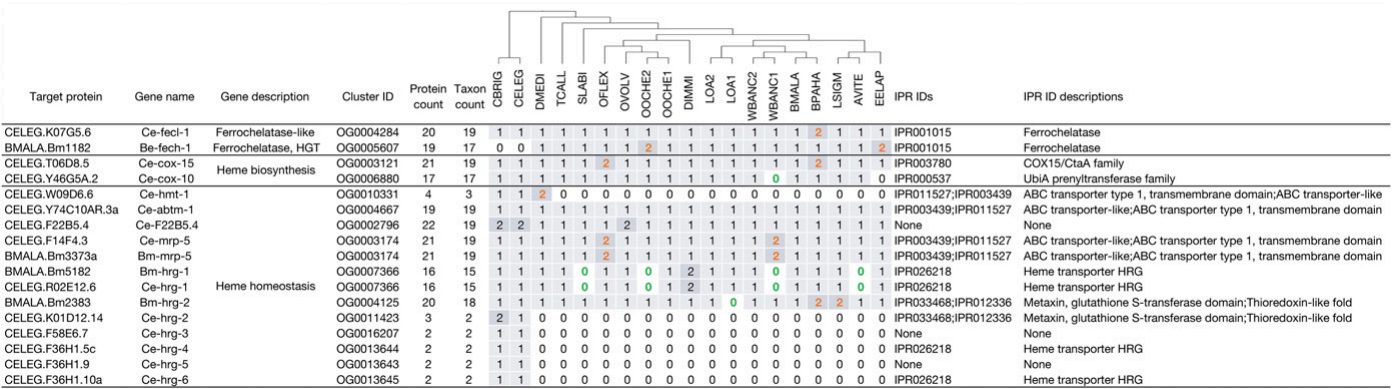
Heme biosynthesis and transport genes. The columns showing counts of proteins per taxon are ordered based on phylogeny (cartoon above). Counts are colored orange if they result from fragmented predictions, and green if the gene was identified only by directed TBLASTN search.

To demonstrate the utility of KinFin for directed analyses of genes and gene families, we revisited the biology of heme synthesis and transport in the Onchocercidae. This pathway is a target of active investigation for drug development. While most organisms can synthesize heme, a complete heme biosynthetic pathway is lacking in all nematodes studied to date (Rao *et al.* 2005), and proteins of only two of 12 catabolic steps (COX-10 and COX-15) have been described in *C. elegans*. In *C. elegans*, multiple heme responsive genes (HRGs) have been characterized (Rajagopal *et al.* 2008; Chen *et al.* 2011; Sinclair and Hamza 2015) and orthologs have been identified in *B. malayi* (Bm-HRG-1 and Bm-HRG-2) and *D. immitis* (Luck *et al.* 2016). In *C. elegans*, HRG are involved in heme trafficking within the epidermis (HRG-2), to oocytes (HRG-3) and within the intestine (HRG-1/4/5/6). Other ABC transporters in *C. elegans* have been implicated in heme homeostasis (MRP-5, F22B5.4, and ABTM-1) (Antonicka *et al.* 2003; Severance *et al.* 2010; González-Cabo *et al.* 2011). An ortholog of MRP-5 has been described in *B. malayi* (Luck *et al.* 2016). Several animal parasitic nematodes (including *B. malayi*, *D. immitis*, and *Onchocerca volvulus*) have been shown to harbor a functional ferrochelatase (FeCH) acquired through horizontal gene transfer from an alphaproteobacterium (Elsworth *et al.* 2011; Nagayasu *et al.* 2013; Wu *et al.* 2013). Other nematodes have distinct ferrochelatase-like (FeCL) homologs that lack the active site. We cataloged homologs of these proteins in our clustering analysis (Figure 6).

FeCL proteins were identified in all species. *Brugia pahangi* has two FeCL proteins (BPAG_0000227101 and BPAG_0000340601-mRNA-1) while all other taxa have one, but both are located at scaffold borders and may be the result of an assembly artifact. The horizontally acquired FeCH is absent from the *Caenorhabditis* proteomes (and genomes) but present in all the other taxa analyzed. Paralogues in one of the *O. ochengi* proteomes (nOo.2.0.1.t09359-RA and nOo.2.0.1.t09412-RA) are suggestive of misprediction. COX-10 and COX-15 are present in most taxa in the analysis; COX-15 paralogs in *B. pahangi* (BPAG_0000577701-mRNA-1 and BPAG_0000682101-mRNA-1), and *O. flexuosa* (OFLC_0000640801-mRNA-1 and OFLC_0000765101-mRNA-1) are a result of fragmented assemblies. COX-10 is present in *W. bancrofti* WBANC1 (on scaffold WBA_contig0009713), but the gene was not predicted. COX-10 was not found in *E. elaphi*, which suggests that either the corresponding genomic region was not assembled, or that the gene has been lost.

Presence/absence of proteins involved in heme homeostasis show a more complex pattern. Ce-HMT-1, an ATP-dependent phytochelatin transporter, is restricted to *Caenorhabditis* spp. and *D. medinensis*. The other ABC-transporter-like proteins (ABTM-1, MRP-5, and F22B5.4) are present across all taxa. For F22B5.4, genuine paralogs are found in both *Caenorhabditis* species and *O. volvulus*. Ce-MRP-5 and Bm-MRP-5 are located within the same cluster, and apparent paralogs in *O. flexuosa* (OFLC_0000058301-mRNA-1 and OFLC_0001303001-mRNA-1) and *W. bancrofti* WBANC1 (WBA_0001142501-mRNA-1 and WBA_0000255201-mRNA-1) derive from predictions located at the ends of scaffolds. While no orthologs of Ce-HRG-2/3/4/5/6 were identified, the cluster containing Ce-HRG-1 included representatives from most species. Missing orthologs of HRG-1 were identified using TBLASTN searches in *S. labiatopapillosa* (nSl.1.1.scaf00038), *O. ochengi* OOCHE2 (nOo.2.0.Scaf03259), *W. bancrofti* WBANC1 (WBA_contig0001821), and *A. viteae* (nAv.1.0.scaf00129). The two HRG-1 paralogs in *D. immitis* were identified previously (Luck *et al.* 2014, 2016). Interestingly, Bm-HRG-2 is not orthologous to Ce-HRG-2 but rather to Ce-C25H3.7, an ortholog of human FAXC (failed axon connection).

In summary, all non-*Caenorhabditis* nematodes analyzed have a functional FeCH orthologous to the one acquired through HGT in *B. malayi*. Proteins responsible for the only two steps in heme biosynthesis described in *C. elegans* are also found in all taxa, apart from COX-10 in *E. elaphi*. The heme transporters HRG-2/3/4/5/6 are (in this analysis) restricted to *Caenorhabditis*, but all the spirurid nematodes analyzed have retained HRG-1, a FAXC-like cluster orthologous to Bm-HRG-2, and MRP-5, and these may mediate heme transport from the intestine.

### Conclusion

As ever more genomes are sequenced, and our understanding of the diversity of protein space increases, it concomitantly becomes more difficult to see the patterns in complex orthology data. To ease this bottleneck, we have presented KinFin—a tool that takes the output of standard orthology inference toolkits, and provides a user-friendly but rich analytical toolkit to review and interrogate orthology clustering. By permitting user definition of custom taxon sets, KinFin can be used to highlight changes in presence or membership of ortholog groups associated with either taxonomy or phenotypes of interest. Its reliance on standard input file formats and explicit parameters makes integration in comparative genomics projects easy, and thus promotes transparent and reproducible analysis of clustered protein data. KinFin is under regular maintenance and we welcome user feedback. Further development will involve a HTML user interface to access and interact with the generated output files.

We presented some of the main capabilities of KinFin through the analysis of proteomes of filarial nematodes and outgroup species. By extracting single-copy orthologs, we resolved the phylogenetic relationships between filarial nematodes. We explored synapomorphic clusters and their functional annotations across the phylogeny and identified putative gene families of interest. Through definition of (phylogenetic) taxon sets, we explored and visualized proteomic diversity across key clades, and analyzed differences in protein counts between the sets. To illustrate targeted analysis of homologs of genes of interest, we analyzed clusters containing proteins involved in heme metabolism and homeostasis using characterized orthologs from model organisms.

One of the advantages of KinFin is the speed of computation. Analyses reported here took 40 sec (basic KinFin analysis) and 4 min (advanced KinFin analysis) on a MacBookPro (15-inch 2016; 2.9 GHz I7, 16 GB RAM). While addition of taxa to the clustering and taxon sets to the config file will increase computation time, we think run time is within acceptable bounds for this type of analysis. KinFin’s speed promotes hypothesis exploration, such as comparing alternative phylogenetic topologies—an example of this has been published recently (Yoshida *et al.* 2017)—or contrasting taxon sets.

## Supplementary Material

Supplemental material is available online at www.g3journal.org/lookup/suppl/doi:10.1534/g3.117.300233/-/DC1.
