# Supplementary material for: KinFin: Software for Taxon-Aware Analysis of Clustered Protein Sequences
Source: G3 (Bethesda). 2017 Sep 1;7(10):3349–57. doi: 10.1534/g3.117.300233 (PMC5633385; doi:10.1534/g3.117.300233)
Supplement: Supplementary file 1 [file 3349FileS1.pdf]

Supplementary methods and results for 'KinFin: Software for taxon-aware analysis of clustered protein sequences'

Dominik R. Laetsch[*,†,1] **and Mark L. Blaxter**[*]

[*]Institute of Evolutionary Biology, University of Edinburgh, Edinburgh EH9 3JT UK
[†]The James Hutton Institute, Errol Road, Dundee DD2 5DA UK

[1]Corresponding author: dominik.laetsch@gmail.com

This document contains the supplementary methods and results.

Supplementary methods

The input and output files, in addition to the commands run, are available on GitHub (`https://github.com/DRL/kinfin_manuscript`).

Clustering and functional annotation of protein sequences

Using `filter_fastas_before_clustering.py`, protein files were filtered by excluding sequences shorter than 30 residues or containing non-terminal stops. Only the longest isoform for each gene was kept by supplying FASTA files and GFF3 annotation to `filter_isoforms_based_on_gff3.py`. Proteins were functionally annotated through `InterProScan v5.22-61.0` (Jones *et al.*, 2014) using the `Pfam-30.0` database (Finn *et al.*, 2016) and converted to KinFin compatible format (`iprs_to_table.py`). `OrthoFinder v1.1.4` (Emms and Kelly, 2015) was used to generate the commands for `BLASTp` analyses. `BLASTp` commands were further modified (`-seg yes`, `-soft_masking true` and `-use_sw_tback`) based on suggestions by Moreno-Hagelsieb and Latimer (2008). `BLASTp` analyses were run on the EDDIE supercomputing cluster at the University of Edinburgh using `BLASTp v2.3.0+` (Camacho *et al.*, 2009). Proteome clustering was carried out at default MCL inflation value of 1.5.

Basic KinFin analysis and phylogenetic inference

An initial KinFin analysis identified 781 single-copy orthologues. Sequences for these 781 clusters were extracted (using the script `get_protein_ids_from_cluster.py` and `GNU grep`) and aligned using `mafft v7.267` (E-INS-i algorithm) (Katoh and Standley, 2013). Alignments were trimmed using `trimal v1.4` (Capella-Gutiérrez *et al.*, 2009), concatenated using `FASconCAT v1.0` (Kück and Meusemann, 2010), and analysed using `RAxML v8.1.20` (Stamatakis, 2014) under the PROTGAMMAGTR model of sequence evolution and 20 alternative runs on distinct starting trees. Non-parametric bootstrap analysis was carried out for 100 replicates.

Advanced KinFin analysis

KinFin was then rerun, providing additional classification in the config file and the functional annotation data. In the config file, taxon sets were defined for the taxonomic rank of 'order' by supplying NCBI TaxIDs for each proteome, for the attribute 'clade' by grouping taxa into taxon-sets for the major filarial clades, and for the attribute 'host' by separating human parasites from those of other animals and outgroups. For the attribute of 'clade', only one proteome per species was allocated to its respective taxon set (*i. e.* LOA2, OOCHE1, and WBANC2) and unique labels were specified for the remaining taxa. The topology of the tree inferred through phylogenetic analysis was provided in Newick format and the two *Caenorhabditis* species were specified as outgroups in the config file. The Mann-Whitney-U test was selected for pairwise protein count representation tests and the required number of proteomes in a taxon-set to be used in rarefaction/representation-test computations was set to 2.

Visualisation of the clustering and calculation of metrics

The distribution of cluster sizes was generated using `plot_cluster_sizes.py` and specifying the colour map 'viridis'. Counts of proteins by cluster type were extracted from `TAXON.attribute_metrics.txt` (folder `TAXON/`).

Representative functional annotation of clusters

Using `get_protein_ids_from_cluster.py`, representative functional annotation (RFA) was inferred for all clusters (`-x 0.75 -p 0.75`, requiring that 75% of proteins in a cluster share a domain and that 75% of proteomes have at least one protein with that domain) and for synapomorphic clusters (`-n 0.75 -x 0.75 -p 0.75`, requiring that also 75% of taxa at a node are present in the cluster).

Analysis of clusters specific to and shared between taxon-sets and assessment of protein space

Analyses on clusters were performed using the following files:

- `order.Rhabditida.cluster_metrics.txt` (folder `order/`)
- `order.Spirurida.cluster_metrics.txt` (`order`)
- `clade.pairwise_representation_test.txt` (`clade`)
- `cluster_counts_by_taxon.txt`

and the representative functional annotation inferred in the previous step. The plot of the rarefaction curves was taken directly from the KinFin output (folder `clade`).

Querying clustering and functional annotation using target genes

The output of KinFin was analysed using the script `get_count_matrix.py` to obtain protein counts by species for genes involved in heme homeostasis and biosynthesis. Presence/absence of unpredicted genes was confirmed through using `TBLASTN v2.3.0+` (Camacho *et al.*, 2009) against the respective genomes. Presence of paralogues was confirmed by manual inspection of gene models on WormBase ParaSite.

Network representation of the clustering

A network representation of the clustering was generated using `generate_network_representation.py` and by ignoring clusters in which all taxa are present (`--exclude_universal`), and visualised using `Gephi v0.9.1` (Bastian *et al.*, 2009). Starting from a random layout, nodes in the graph were positioned using the force directed ForceAtlas2 layout algorithm (Jacomy *et al.*, 2014) using the parameters: `Tolerance = 0.2`, `Approximation = 1.2`, `Approximate Repulsion = False`, `Scaling = 5000`, `Stronger Gravity = False`, `Gravity = 1.2`, `LinLog mode = True`, `Dissuade hubs = True`, `Prevent overlap = True`, `Edge Weight Influence = 1.0`. Under this layout algorithm nodes repulse each other like charged particles, while edges attract their nodes like springs. Nodes were coloured by phylogenetic clade and scaled proportional to the size of the proteome.

Comparison of clustering behaviour of proteomes for which two assemblies exist

Clustering behaviour was analysed by consulting the `TAXON.*.cluster_metrics.txt` files for each of the proteomes in question.

Supplementary results

Network representation of the clustering

The network (Figure S1) showed no consistent phylogenetic patterning between the taxa, with exception of the separation of the orders Rhabditida (grey) and Spirurida (coloured). Positioning of individual nodes within Spirurida varied for different runs of the layout algorithm (data not shown) and even nodes of the same species (*i. e.* LOA1/LOA2, WBANC1/WBANC2, OOCHE1/OOCHE2) were occasionally, spatially separated in the network which indicated non-overlapping gene predictions between the different proteomes for a given species.

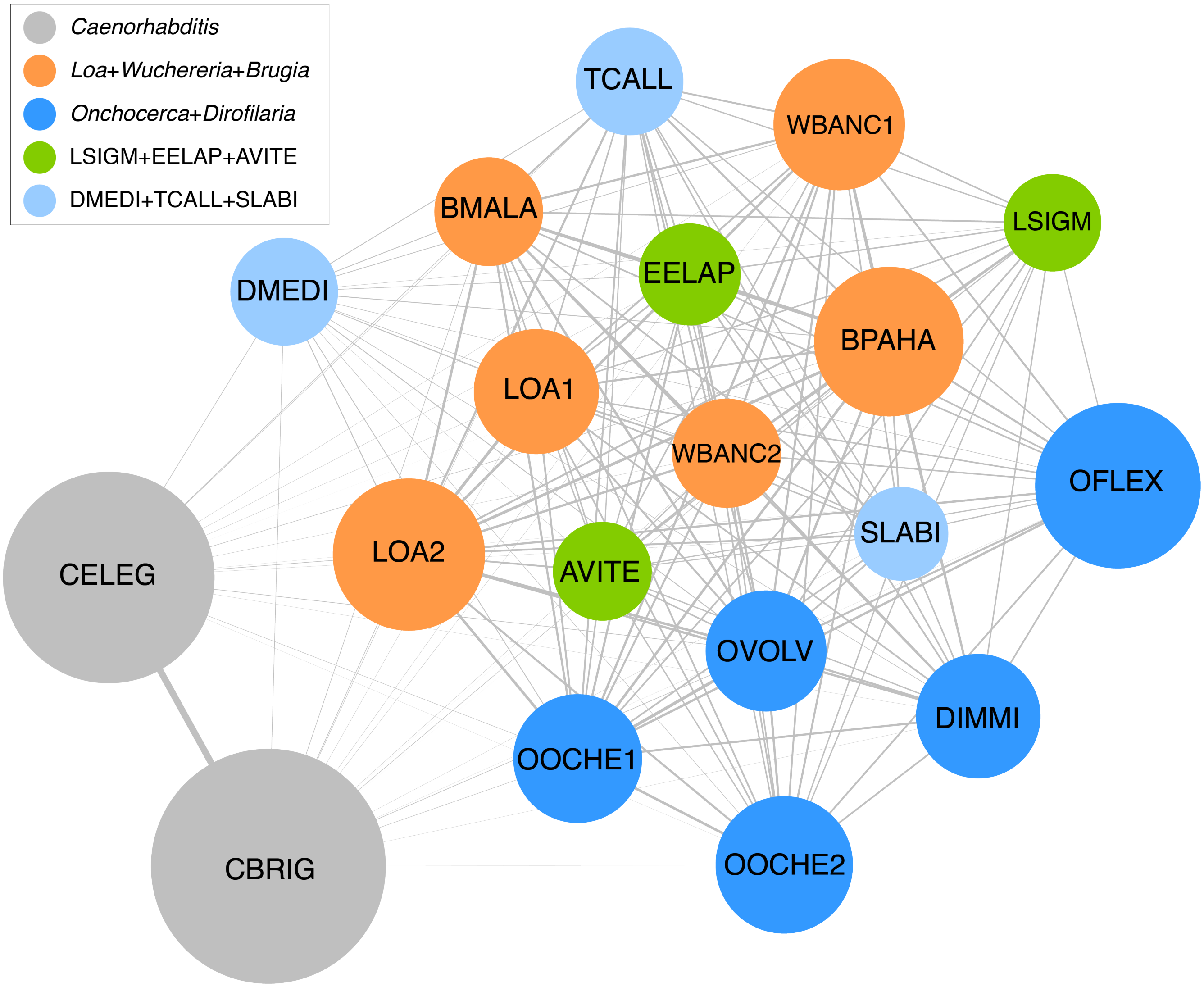

Figure 1. Network representation of the clustering data. Proteomes are represented by nodes, coloured by based on phylogenetic clade, scaled by count of proteins, and positioned by a force directed layout algorithm. Edges are drawn between two nodes, weighted by the number of clusters in which both proteomes occur simultaneously (excluding clusters in which all proteomes are present)

Comparison of clustering behaviour of proteomes for which two assemblies exist

The different clustering behaviour for the proteins predicted for these assemblies is shown in Figure S2. For *L. loa* and *W. bancrofti*, higher number of proteins in a proteome correlates with both higher number of singleton proteins and proteins in clusters shared with other nematode species. Interestingly, genome assemblies for *W. bancrofti* differ substantially in contiguity and CEGMA completeness (see WormbaseParasite) and the higher proportion of proteins that WBANC1 shares with other species might be a result of fragmented gene predictions, since the average mean lengths of WBANC2 proteins in shared

clusters is 91 residues shorter than WBANC1 proteins (436 versus 345).

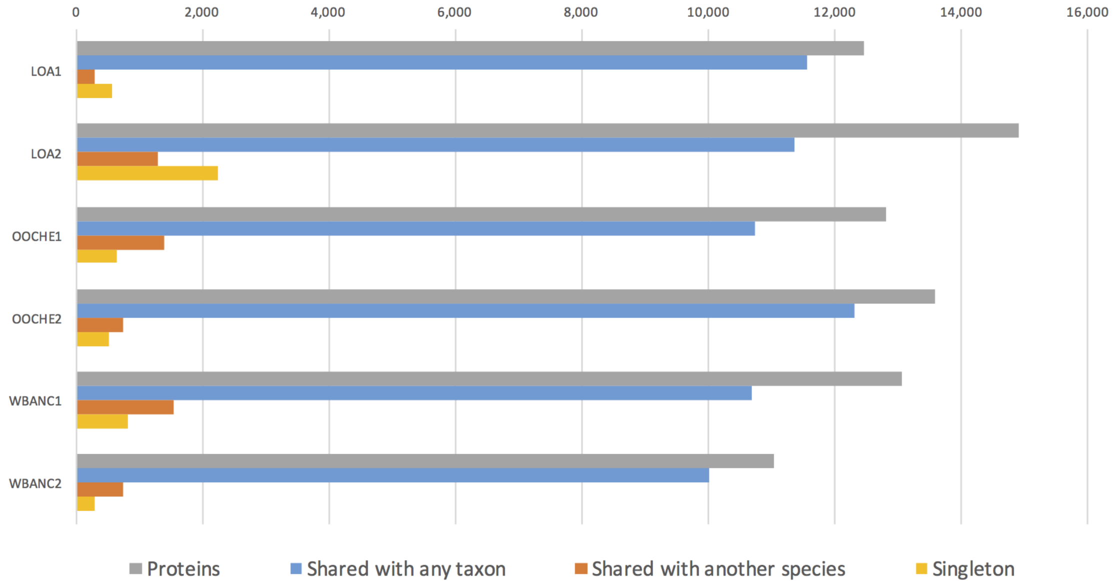

Figure 2. Count of proteins for proteomes of the same species. Proteins: total number of proteins. Singleton: number of proteins in singleton clusters. Shared with another species: proteins in clusters shared with another nematode species. Shared with any taxon: proteins in clusters shared with any taxon.

References

Bastian, M., S. Heymann, and M. Jacomy, 2009 Gephi: An open source software for exploring and manipulating networks. In *International AAAI Conference on Weblogs and Social Media*.

Camacho, C., G. Coulouris, V. Avagyan, N. Ma, J. Papadopoulos, *et al.*, 2009 Blast+: architecture and applications. BMC Bioinformatics **10**: 421.

Capella-Gutiérrez, S., J. M. Silla-Martínez, and T. Gabaldón, 2009 trimal: a tool for automated alignment trimming in large-scale phylogenetic analyses. Bioinformatics **25**: 1972–1973.

Emms, D. M. and S. Kelly, 2015 Orthofinder: solving fundamental biases in whole genome comparisons dramatically improves orthogroup inference accuracy. Genome Biol **16**: 157.

Finn, R. D., P. Coggill, R. Y. Eberhardt, S. R. Eddy, J. Mistry, *et al.*, 2016 The pfam protein families database: towards a more sustainable future. Nucleic Acids Res **44**: D279–85.

Jacomy, M., T. Venturini, S. Heymann, and M. Bastian, 2014 Forceatlas2, a continuous graph layout algorithm for handy network visualization designed for the gephi software. PLoS ONE **9**: e98679.

Jones, P., D. Binns, H.-Y. Chang, M. Fraser, W. Li, *et al.*, 2014 Interproscan 5: genome-scale protein function classification. Bioinformatics **30**: 1236–1240.

Katoh, K. and D. M. Standley, 2013 Mafft multiple sequence alignment software version 7: improvements in performance and usability. Mol Biol Evol **30**: 772–780.

Kück, P. and K. Meusemann, 2010 Fasconcat: Convenient handling of data matrices. Mol Phylogenet Evol **56**: 1115–8.

Moreno-Hagelsieb, G. and K. Latimer, 2008 Choosing blast options for better detection of orthologs as reciprocal best hits. Bioinformatics **24**: 319–324.

Stamatakis, A., 2014 Raxml version 8: a tool for phylogenetic analysis and post-analysis of large phylogenies. Bioinformatics **30**: 1312–1313.
